# Editorial: “Omics”- revolution in elucidating the virulence and resistance in *Staphylococcus aureus*

**DOI:** 10.3389/fcimb.2023.1209671

**Published:** 2023-05-17

**Authors:** Dennis Nurjadi, Jan Tkadlec, Sébastien Boutin, François Vandenesch

**Affiliations:** ^1^ Department of Infectious Diseases and Microbiology, University of Lübeck, Lübeck, Germany; ^2^ Department of Medical Microbiology, Charles University, 2nd Faculty of Medicine and Motol University Hospital, Prague, Czechia; ^3^ CIRI, Centre International de Recherche en Infectiologie, Université de Lyon, Inserm, U1111, Université Claude Bernard Lyon 1, Centre National de la Recherche Scientifique (CNRS), UMR5308, École Normale Supérieure (ENS) de Lyon, Lyon, France; ^4^ Centre National de Référence des Staphylocoques, Institut des Agents Infectieux, Hospices Civils de Lyon, Lyon, France

**Keywords:** *Staphylococcus aureus*, omics, USA300, panton-valentine leukocidin, resistance and virulence

*Staphylococcus aureus* is one of the most common causes of bacterial infections in humans, and the bacterial pathogen with the highest number of attributable deaths (Collaborators, 2022). The human nasal cavity serves as the natural reservoir for *S. aureus*. Although many healthy individuals are colonized with *S. aureus*, only some develop infections (Wertheim et al., 2005). The transition from a commensal to an opportunistic pathogen is not fully understood, but virulence factors likely play a significant role in initiating and facilitating infection processes (Howden et al., 2023). This Research Topic features articles showcasing the application of cutting-edge molecular biology methods (“Omics”) to elucidate the virulence and resistance of *S. aureus*.

The convergence of resistance and virulence is an intriguing phenomenon increasingly observed in many bacterial species (Li et al., 2021; Biggel et al., 2022). One of the archetypes of this convergence is the emergence of virulent community-associated methicillin-resistant *S. aureus* (MRSA), USA300 strains (Nimmo, 2012).To shed light on the events that shaped the evolution of this lineage, Bianco et al. investigated the evolution of the pandemic MRSA strain USA300 by analyzing and comparing genomic sequences of circulating USA300 strains and USA300 strains that predate the dominance of this expansive clone. They notably uncovered a pre-epidemic branching clade consisting of (already Panton-Valentine leukocidin (PVL)-positive) both methicillin-susceptible *S. aureus* (MSSA) and MRSA isolates circulating around the world that diverged from the USA300 lineage prior to the establishment of the South American and North American epidemics.

The treatment of infections caused by *S. aureus* can be challenging, as recurrences or chronicity may occur despite appropriate therapy (Tuchscherr et al., 2020). The study by Klein et al. found that even when belonging to the same clone, *S. aureus* isolated from different body sites and infection foci may exhibit differences in virulence and resistance phenotypes. This phenotypic plasticity and heterogeneity can be attributed to the integration of Sa3int bacteriophages into the β-hemolysin (*hlb*) gene, which results in the truncation of the *hlb* gene and the insertion of genes encoding staphylokinase (*sak*) and staphylococcal complement inhibitor (*scn*), leading to a highly plastic immune evasive phenotype.

Blocking bacterial virulence to promote pathogen killing and elimination by the immune system is an interesting alternative treatment approach (Ford et al., 2020). The study by Zhou et al. investigated the role of small RNA SprC using RNASeq for transcriptomics analysis on the metabolism and virulence of *S. aureus* N315. Over 2,497 identified transcripts, the SprC-mutant N315 *S. aureus* exhibited 23 downregulated differentially expressed genes, mainly related to metabolism and pathogenesis. Considering the emergence of drug resistance in *S. aureus*, such “pathoblockers” may be a promising alternative treatment strategy.

Traditionally, the clinical severity of *S. aureus* infections is associated with the presence or absence of certain genes coding some of the various *S. aureus* virulence factors (Howden et al., 2023). However, the impact of the expression levels of these virulence factors has been underexplored, largely due to the lack of high-throughput quantification methods for virulence proteins. In the study conducted by Pivard et al., the authors investigated the quantitative virulomes of 136 *S. aureus* isolates using a targeted proteomic approach. Their findings revealed that several virulence factors, including PVL, were associated with severity parameters in a dose-dependent manner, providing the proof of concept that “expression matters” in pathogen virulence and can be inferred from *in vitro* culture of the corresponding strain.

Nasal colonization with *S. aureus* is associated with an increased propensity to acquire infections (Bode et al., 2010). Therefore, understanding the mechanisms of persistent nasal colonization may help identify novel targets and strategies to decolonize high-risk patients. In their study, Salgado et al. used serial passaging of a murine colonization model and genome sequencing to demonstrate that changes were found in genes associated with the cell surface and metabolism, which might indicate niche adaptation in *S. aureus* to promote long-term colonization.

The articles presented in this Research Topic showcase the promising use of “OMICs” technologies in advancing research on *S. aureus* virulence and resistance. Specifically, the application of transcriptomics and proteomics adds a new functional and mechanistic dimension to elucidating the pathophysiology of *S. aureus* infections. By gaining a deeper understanding of the correlation between virulence factors and clinical outcomes, we may be able to improve diagnostic and therapeutic strategies for *S. aureus* infections.

## Author contributions

All authors listed have made a substantial, direct, and intellectual contribution to the work and approved it for publication.
